# Enhancing the mechanical properties and cytocompatibility of magnesium potassium phosphate cement by incorporating oxygen-carboxymethyl chitosan

**DOI:** 10.1093/rb/rbaa048

**Published:** 2020-12-03

**Authors:** Changtian Gong, Shuo Fang, Kezhou Xia, Jingteng Chen, Liangyu Guo, Weichun Guo

**Affiliations:** Department of Orthopedics, Renmin Hospital of Wuhan University, Wuhan 430060, China

**Keywords:** magnesium phosphate cement, oxygen-carboxymethyl chitosan, physicochemical properties, cytocompatibility

## Abstract

Incorporating bioactive substances into synthetic bioceramic scaffolds is challenging. In this work, oxygen-carboxymethyl chitosan (O-CMC), a natural biopolymer that is nontoxic, biodegradable and biocompatible, was introduced into magnesium potassium phosphate cement (K-struvite) to enhance its mechanical properties and cytocompatibility. This study aimed to develop O-CMC/magnesium potassium phosphate composite bone cement (OMPC), thereby combining the optimum bioactivity of O-CMC with the extraordinary self-setting properties and mechanical intensity of the K-struvite. Our results indicated that O-CMC incorporation increased the compressive strength and setting time of K-struvite and decreased its porosity and pH value. Furthermore, OMPC scaffolds remarkably improved the proliferation, adhesion and osteogenesis related differentiation of MC3T3-E1 cells. Therefore, O-CMC introduced suitable physicochemical properties to K-struvite and enhanced its cytocompatibility for use in bone regeneration.

## Introduction

Because thousands of individuals suffer from bone defects caused by various orthopedic diseases, the reconstruction of bone defects represents a critical challenge worldwide [1, 2]. Traditional methods, especially for autografts and allografts, which associate with the risk of disease transfer, the possibility of immunogenicity and an increase in operation cost, cannot adequately meet clinical demands [3]. Encouragingly, synthetic biomaterials have been considered ideal alternatives to address the issues mentioned above [4]. 

Bone cement is the most commonly used synthetic biomaterial in the area of bone repair [5]. Poly(methyl methacrylate) (PMMA) cement, which is characterized by suitable mechanical properties and easy mass production, has been used for minimally invasive surgery since the 1930s [6]. Unfortunately, some disadvantages of this cement, including low bioactivity, the toxicity of monomers and an exothermic reaction during polymerization, restrict its further development [7]. Compared with PMMA cement, calcium phosphate cement (CPC) has been commonly used owing to its prominent biocompatibility and osteoconductivity, as well as its resemblance to the inorganic phase of bone [8, 9]. However, CPCs have two fatal disadvantages: their application for bone reconstruction is mainly restricted by a low rate of bioresorption, and they have relatively poor mechanical properties, which is a major concern regarding their clinical and which allows them to be used only in non-load-bearing areas [10].

Magnesium phosphate cements (MPCs) have recently been approved as potentially ideal substitutes for CPCs since an efficient combination of relatively high mechanical properties and faster bioresorption rates was achieved [11]. This subtype of bone cement is obtained from a quick acid−base reaction; a viscous paste is formed upon mixing the solid and liquid phase together. This reaction is a chemical dissolution reaction, where the entanglement of the crystals in the paste leads the self-setting of bone cement [12−14]. Moreover, recent studies have found that the proper release of magnesium ions during MPC degradation can enhance the bioactivity of osteoblasts [15]. Unfortunately, the ammonia emission during struvite preparation could restrict its clinical application range. Therefore, some researchers have utilized potassium dihydrogen phosphate (KH_2_PO_4_) as a more suitable phosphate source to avoid ammonia emission, with the final hydration product regarded as magnesium potassium phosphate cement (K-struvite) [16, 17]. However, the critical problem with the K-struvite system is achieving proper control of the fast self-setting speed [18]. Thus, common modifications to a basic cement system are of great necessity in the biomedical applications of K-struvite. Changes to reaction formulas may contain a combination of different systems, typically achieved through the addition of retardants. Retardants, such as chitosan (CS) and other bioactive substances, have been added to slow the reaction speed of the K-struvite system [11].

The incorporation of bioactive substances is considered to be an valid method to enhance the biological properties of bone cement and control its hydration rate at the same time [19]. CS, as a nontoxic, biocompatible and biodegradable natural polysaccharide, has been proverbially known as an additive for orthopedic bioengineering applications [20]. However, the application of CS in the MPC system is severely limited owing to its steady crystal structure, which limits its solubility in alkaline pH [21]. To solve this vital issue, an *O*-carboxymethyl derivative of CS (O-CMC) was selected as an ideal substitute in the preparation of composite MPC scaffolds [22]; O-CMC has been indicated to have the ability to facilitate the proliferation and differentiation of osteoblasts. Dou *et al.* successfully used O-CMC as a drug carrier to construct a rHBMP-2 and VEGF two-factor compound sustained-release system. The results revealed that this composite scaffold could facilitate the formation of blood vessels and the proliferation and differentiation of preosteoblasts *in vivo* [23]. Therefore, it was indicated that O-CMC might serve as a promising additive acting as retarding agent in K-struvite system for bone regeneration.

This work aimed to modify K-struvite with a proper physicochemical property and good cytocompatibility using dead-burned MgO, KH_2_PO_4_ and different contents of O-CMC. No previous studies have focused primarily on evaluating the effect of O-CMC on the properties of K-struvite. We hypothesized that the addition of O-CMC could slow the hydration speed and increase the mechanical properties of K-struvite. We also expected an appropriate amount of O-CMC to facilitate osteogenic proliferation, adhesion and differentiation.

## Materials and methods

### Preparation of OMPC scaffolds

K-struvite original raw materials were formed by setting liquid and powder phase. Dead-burnt magnesium oxide (MgO) was uniformly mixed with potassium dihydrogen phosphate (KH_2_PO_4_) at a parameter of 1.3:1 (molar ratio). The initial light MgO was calcined at 1600°C for 6 h and next ground by planetary ball mill for 15 min. KH_2_PO_4_ was also milled for 30 min using the same planetary ball mill. Along with dead-burnt MgO and KH_2_PO_4_, 0, 1, 2.5 and 5 wt.% O-CMC powders were added to prepare the O-CMC-loaded K-struvite powder phase. Deionized water was used as setting liquid, and the powder to liquid ratio used was 2 g/ml. According to the differences in the content of O-CMC, the collected self-setting scaffolds were named OMPC-0, OMPC-1, OMPC-2.5 and OMPC-5; all the formulas are shown in Table 1.

**Table 1.** Composition of OMPC scaffolds

Sample	MgO: KH_2_PO_4_ (molar ratio)	O-CMC addition(wt.%)	Liquid phase	P/L ratio （g/ml）
OMPC-0	1.3:1	0	Deionized water	2
OMPC-1	1.3:1	1	Deionized water	2
OMPC-2.5	1.3:1	2.5	Deionized water	2
OMPC-5	1.3:1	5	Deionized water	2

O-CMC, oxygen-carboxymethyl chitosan.

### Characterization of OMPC scaffolds

The phases of the OMPC scaffolds after 48 h standard curing condition (37°C and 100% humidity) were characterized by X-ray diffraction (XRD; XPert Pro, PANalytical, the Netherlands) using Cu-Kα radiation, and data were collected for 2θ (10°−80°) with a scan rate of 4°/min and at a step size of 0.013°. The surface morphologies of the hydrated scaffolds after 48 h standard curing condition were analyzed using a field emission scanning electron microscope (FESEM; Zeiss SIGMA, Carl Zeiss, Germany). Fourier transform infrared (FTIR) spectra were collected through an infrared spectroscope (NICOLET 5700 FTIR Spectrometer, Thermoelectron Scientific Instruments, USA) using scaffolds in the form of pellets formed with spectroscopic grade potassium bromide for chemical band analysis.

### Setting time, compressive strength and porosity of OMPC scaffolds

According to GB/T1346-2001, a Vicat apparatus was set to measure the initial and final setting time of OMPC scaffolds. After adding the powder to the liquid phase, the mixture was immediately transferred to a mold (Φ15 × 8 mm). The initial setting time was marked as the phase from the time of blending two phases together to the time when the Vicat apparatus needle was no <5 mm from the bottom. For the final setting time, recording time point is that Vicat apparatus needle was able to penetrate the scaffold by <1 mm. Each experiment was repeated three times.

Per ISO13779-1, the compressive strength of OMPC scaffolds (Φ6 mm ×12 mm) after 48 h standard curing condition was tested using a universal material testing machine (RGM4100, REGER Industrial Systems, China) at a loading rate of 2 mm/min. Each formula was measured five times.

The porosity of the prepared OMPC scaffolds (Φ6 mm ×12 mm) after 48 h standard curing condition was determined using a method of immersion. The scaffolds were soaked in anhydrous ethanol until saturated. Next, the scaffold that weighted before and after soak were named M0 and M1, respectively. The suspended weight of the anhydrous ethanol-soaked scaffold was named M2. All scaffolds were triplicated in the experiment, and the porosity was counted by the formula:

$$\mathrm{Porosity}\;\left(\%\right)=\frac{\left(M_{1}-M_{0}\right)}{\left(M_{1}-M_{2}\right)}\times 100$$

### *In vitro* degradation, pH variation and Mg^2+^ release of OMPC scaffolds

The weight loss ratios of the OMPC samples (Φ6 mm ×12 mm) were measured at different time after being hydrated for 48 h under standard curing condition. $W_{0}$ was recorded as the initial dry weight, and $W_{t}$ was recorded as the dry weight at time point *t*. The scaffolds were soaked in Tris–HCl solution (pH = 7.4) at a weight-to-volume ratio of 200 mg/ml and continually shaken for 28 days in a shaking bath. The Tris–HCl solution was replaced every other day. The specimens were removed on the 1, 3, 7, 14, 21 and 28 day; washed with deionized water; and then dried at 60°C for 2 h. All the values recorded were average values of three specimens. The weight loss ratio was collected by the subsequent equation:

$$\mathrm{Weight}\;\mathrm{loss}\;\left(\%\right)=\frac{\left(W_{0}-W_{t}\right)}{W_{0}}\times 100.$$

The pH variation of the Tris–HCl solution after soaking for 28 days was measured using pH meter (PHS3C, Yueping, China) at the same time point as above. Three samples were measured in parallel each day.

The OMPC scaffolds were cultured in Tris–HCl solution for 72 h with no Tris–HCl solution replacement, and variations of Mg^**2+**^ concentration were recorded using inductively coupled plasma-atomic emission spectroscopy (ICP-AES, Prodigy 7, LEEMAN LABS, USA) after 24 and 72 h.

### Cell viability and morphology assay

The *in vitro* cell viability of the OMPC samples was obtained by the Cell Counting Kit 8 (CCK-8, Beyotime, China) as described below. OMPC scaffolds (Φ4 mm ×2 mm) for the cell experiment were sterilized using ethylene oxide, next placed in a ventilated environment for gas removal. Next, 5 × 10^4^/ml MC3T3-E1 cells were seeded in 96-well plates and cultured with OMPC scaffolds mentioned above. Sterilized cement disks were mixed with α-MEM complete cell culture medium (10% fetal bovine serum, 1% penicillin and streptomycin) at a ratio of 180 mg/ml. The plates were then incubated at a standard condition (37°C and 100% humidity with 5% CO_2_) in an incubator; α-MEM complete cell culture medium was replaced every 2 days. Pure α-MEM complete culture medium served as a control. On the 1st, 3rd and 5th day, 10 μl of a CCK-8 solution was added to each well. Before testing, the disks were removed, and the 96-well plates were then incubated in a cell culture incubator for 2 h, after which their emissions were read at 450 nm using a microplate reader (ELX080, PerkinElmer, USA). Each group consisted of five samples, and the experiment was repeated three times.

Following incubate for 1, 3 and 5 days, MC3T3-E1 cells cultured with OMPC scaffolds were washed by phosphate-buffered saline solution (PBS), and then using a 4% paraformaldehyde to fix for 15 min, then washed again with PBS solution, and the nuclei and cytoskeletons were respectively stained with 4′-6-diamidino-2-phenylindole (DAPI, 100 ng/ml) and fluorescein isothiocyanate (FITC)-labeled phalloidin (100 nM). The MC3T3-E1 cells morphology was observed by confocal microscopy (C2, Nikon, Japan).

### Cell attachment experiment

The MC3T3-E1 cells morphology and adhesion to OMPC scaffolds surface were evaluated. First, 4 − 5 × 10^3^ MC3T3-E1 cells in 75 μl of culture medium were seeded on sample surfaces (Φ6 mm × 2 mm), and these cell-containing samples were incubated at a standard condition (37°C, 100% humidity and 5% CO_2_) for 3 and 5 days. Then 1 wt.% glutaraldehyde solution was used to fix MC3T3-E1 cells, followed by rinsing of the cells with different grades of alcohol, i.e. 50, 60, 70, 80, 90, 95 and 100. Finally, cell-containing OMPC scaffolds were painted with gold and observed using FESEM (Zeiss SIGMA).

### Osteogenic differentiation assay

A quantitative real-time polymerase chain reaction (qRT-PCR) assay was used to measure the osteogenic differentiation of the MC3T3-E1 cells cultured with the OMPC scaffolds. After culturing MC3T3-E1 cells with OMPC scaffolds (Φ4 mm ×2 mm) in 3 × 10^4^ cells/well for 7 and 14 days, the cellular RNA was taken out with TRIzol reagent (Invitrogen). Then, cDNA was obtained by the PrimeScript RT reagent Kit (Takara, Tokyo, Japan). Next, 4 ml 10-fold diluted cDNA aliquot was submitted to real-time PCR by SYBR Premix Ex Taq™. Real-time PCR was represented using a Bio-Rad real-time PCR system (Bio-Rad, Hercules, CA, USA) with referents of alkaline phosphatase (ALP), collagen type I (COL-1), osteocalcin (OCN) and Runt-related transcription factor (Runx2), with glyceraldehyde-3-phosphate dehydrogenase set as housekeeping gene. The relative expression level for each osteogenic differentiation-related target gene was determined using the 2^−ΔΔCt^ method. The primers employed in this study are shown in Supplementary Table S1.

### Statistical analysis

All data are expressed as the mean ± standard deviation (mean ± SD). Statistical analysis was carried out using one-way ANOVA followed by Tukey’s post hoc test with a statistical significance at *P* < 0.05.

## Results

### Setting time, compressive strength and porosity of OMPCs

**Figure 1.**
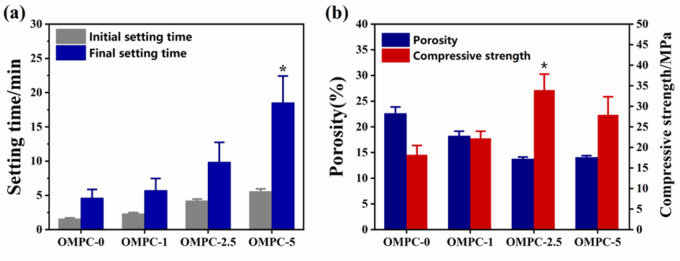
(**a**) Effect of O-CMC on the setting time of OMPCs. (**b**) The porosity and compressive strength were determined after 48 h standard curing condition (37°C and 100% humidity) (*P < 0.05 vs. OMPC-0)

The effect of O-CMC on the setting time of OMPC scaffolds is seen in Fig. 1a. Both the initial and final setting times prolonged with increasing concentrations of O-CMC. For OMPC-5, the final setting time increased to 18 min, which was significantly different than the setting time of OMPC-0 of 4 min (*P* < 0.05). The effects of O-CMC on the porosity and compressive strength are exhibited in Fig. 1b. The porosity of OMPC scaffolds decreased with increasing O-CMC amount. For OMPC-0, the porosity of the scaffold was 22.6%. For OMPC-2.5, the porosity was 13.8%, which was the lowest value observed. The compressive strength increased with increasing O-CMC content, and OMPC-2.5 maximize to 33.8 MPa (*P* < 0.05). Nevertheless, the compressive strength decreased with a further increase in the O-CMC content.

### Characterization of OMPC paste

**Figure 2.**
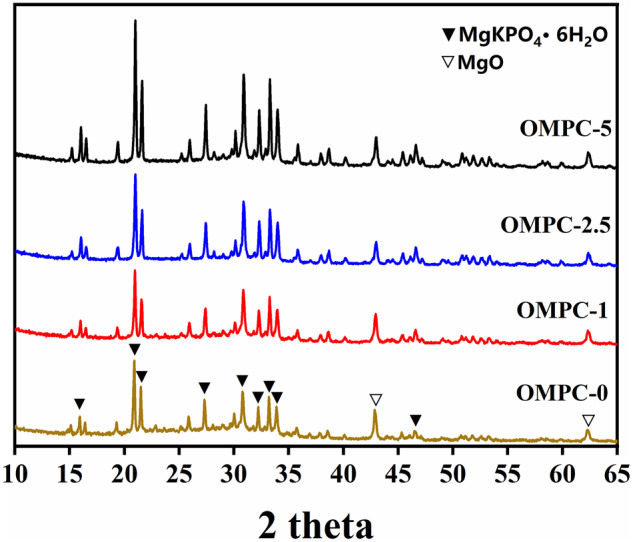
XRD patterns of OMPC scaffolds after 48 h standard curing condition (37°C and 100% humidity)

As shown in Fig. 2, the XRD data showed typical MgKPO_4_·6H_2_O peaks and peaks of unreacted MgO. The highest crystallinity of MgKPO_4_·6H_2_O was observed in OMPC-5, followed by OMPC-2.5, OMPC-1 and OMPC-0. However, the diffraction peaks of MgO among all OMPC groups showed no significant difference.

**Figure 3.**
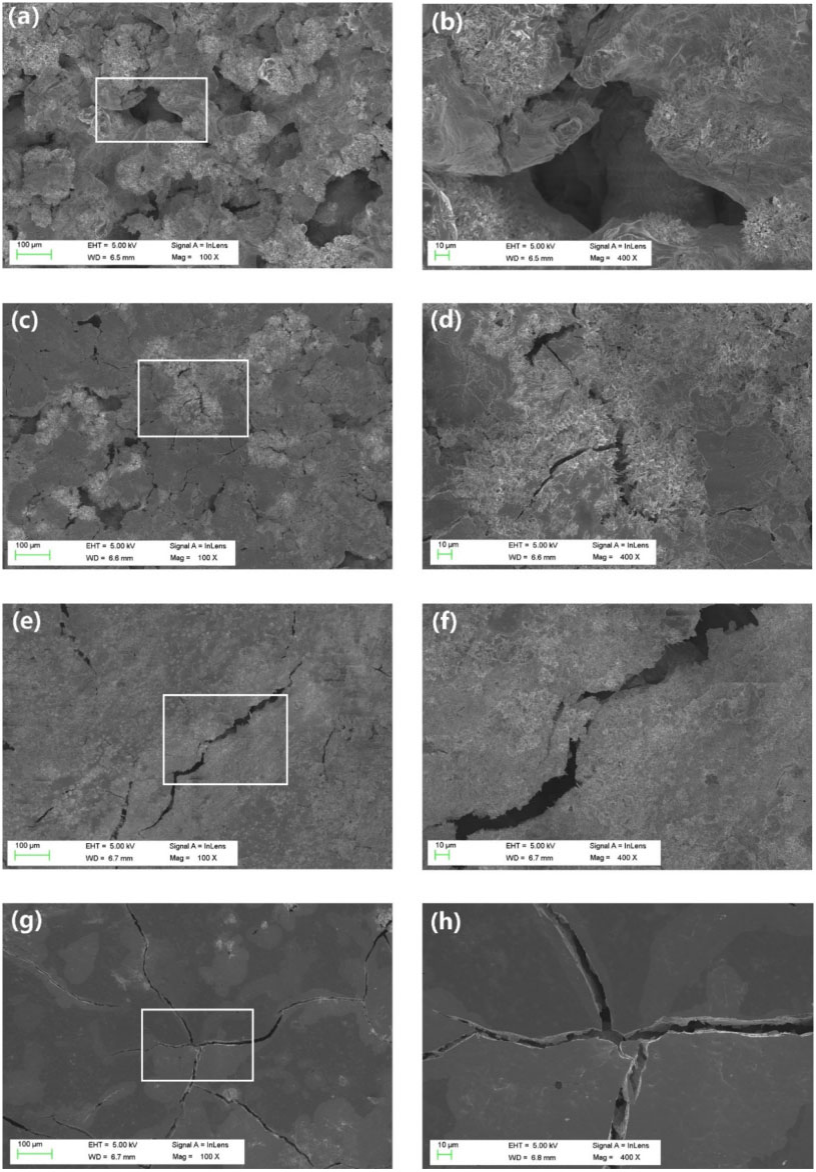
FESEM images of OMPC-0 (**a**, **b**), OMPC-1 (**c**, **d**), OMPC-2.5 (**e**, **f**) and OMPC-5 (**g**, **h**) after 48 h standard curing condition (37°C and 100% humidity).

FESEM images of the OMPCs are presented in Fig. 3. OMPC-0 was composed of fragmented and clustered MgKPO_4_·6H_2_O crystals, irregular porous structures and microcracks, indicating its high brittleness. For OMPC-1, OMPC-2.5 and OMPC-5, the irregular porous structures gradually disappeared, and fewer fragmented MgKPO_4_·6H_2_O crystals as well as small cracks, which became increasingly longer, were observed.

**Figure 4.**
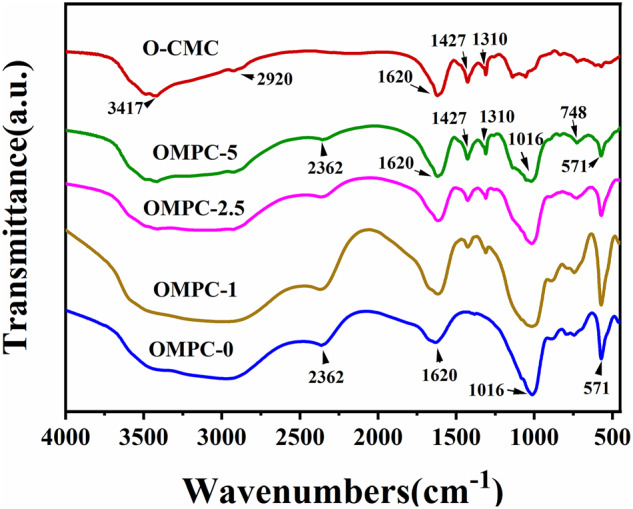
FT-IR Spectra of the OMPCs after 48 h standard curing condition (37°C and 100% humidity).

The FT-IR spectra of the pure O-CMC and the OMPCs are shown in Fig. 4. The absorption bands observed at 1620.2/cm (N−H), 1427.2/cm (−CH) and 1310.3/cm (−CH) were attributed to the characteristic carboxymethyl groups from pure O-CMC. The spectra of all OMPCs except OMPC-0 showed the characteristic absorption bands at 1427.2/cm (−CH) and 1310.3/cm (−CH). Moreover, the stretching vibration absorption peaks at 1427.2/cm (−CH) and 1310.3/cm (−CH) increased in intensity with increasing amounts of O-CMC.

### *In vitro* variation of weight loss, pH and Mg^2+^ release of OMPCs

**Figure 5.**
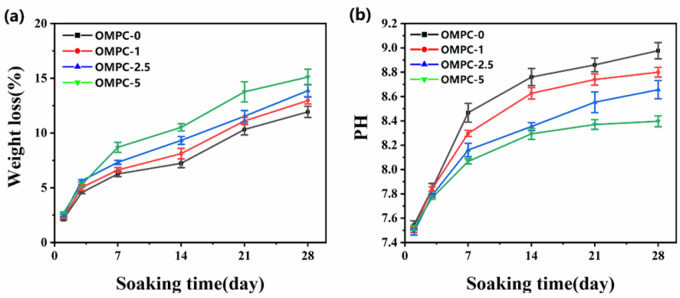
OMPCs weight loss (**a**) and pH variation (**b**) during soaking in Tris−HCl solution for 28 days.

Figure 5a represents the *in vitro* weight loss ratio of the OMPC scaffolds immersed in Tris–HCl solution. All OMPC groups visibly degraded in the Tris–HCl solution over time, and two main stages of *in vitro* degradation were observed. In the first 3 days, ∼5% of the total weight of all OMPC groups was lost, and the fastest degradation rate occurred during this phase. During the next 25 days, 6 − 10% of the total weight was lost, and the weight loss ratio reached 15.11 wt.% for OMPC-5. Figure 5b indicates that the pH of all OMPC groups was alkaline and gradually increased over 28 days. Day 7 marked a turning point in the pH change, after which it increased at a lower rate. Moreover, the incorporation of O-CMC slightly decreased the final pH of the OMPCs.

The changes in Mg^**2+**^ concentrations of in the Tris–HCl solutions after soaking are shown in Table 2. The Mg^**2+**^ concentration in the Tris–HCl solution increased over a 72 h time period for all OMPC groups. In the first 24 h, the Mg^**2+**^ concentration in the OMPC-5 sample was remarkably higher than that of other OMPC groups (*P* < 0.05). However, during the next 48 h, no significant difference was defined in the ion concentrations of Mg^**2+**^ for the different groups. In addition, for OMPC-5, ∼50% of Mg^**2+**^ was released during the first 24 h.

**Table 2.** Concentration of Mg^2+^ in the Tris–HCl solution of OMPCs (mg/l)

Time (h)	OMPC-0	OMPC-1	OMPC-2.5	OMPC-5
24	9.8 ± 2.3	10.2 ± 2.7	13.5 ± 1.8	21.4 ± 2.9^*^
72	33.8 ± 4.2	35.2 ± 4.6	39.6 ± 3.4	44.7 ± 5.2

* *P* < 0.05.

### *In vitro* cytocompatibility

**Figure 6.**
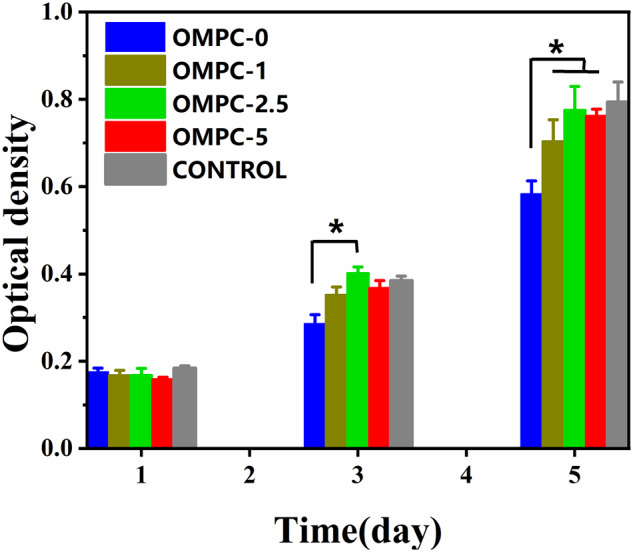
Proliferation MC3T3-E1 cells incubated with OMPC scaffolds for 1, 3 and 5 days. *Significant difference at *P* < 0.05 vs. OMPC-0.

The proliferation of MC3T3-E1 cells cultured with OMPC scaffolds was obtained using the CCK-8 assay. Figure 6 shows no significant difference after 1 day for all OMPC groups; after 3 days, the optical density value for OMPC-2.5 was significantly higher than that for OMPC-0 (*P* < 0.05). However, OMPC-1, OMPC-2.5 and OMPC-5 showed a higher cytocompatibility than OMPC-0 after 5 days (*P* < 0.05). Compared with OMPC-0, the results showed that OMPC-1, OMPC-2.5 and OMPC-5 could facilitate cell proliferation, which is consistent with the control group.

**Figure 7.**
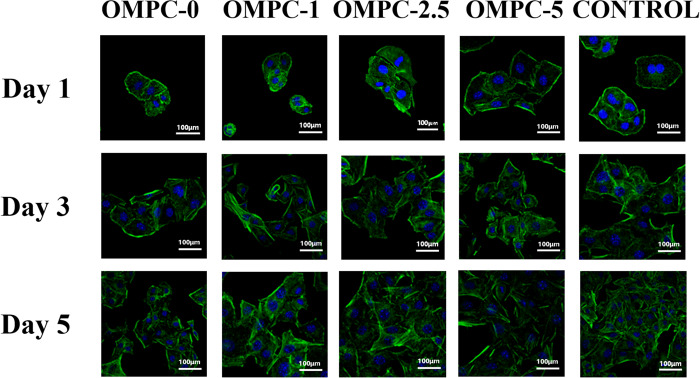
Confocal laser scanning microscope pictures of MC3T3-E1 cells incubated with OMPC scaffolds for 1, 3 and 5 days. Nuclei (blue) were stained with DAPI and F-actin cytoskeleton (green) were stained with FITC-phalloidin.

The morphology of the cells cultured with different OMPC scaffolds after 1, 3 and 5 days was analyzed by fluorescence staining. As exhibited in Fig. 7, none of the OMPC scaffolds had an adverse effect on the cell morphology, viability or proliferation. After 3 days of culturing, the cells stretched sufficiently without showing any abnormal morphology. On the 5th day, cells cultured with OMPC-2.5 had a higher density and were more widely distributed than those cultured with OMPC-0, OMPC-1 and OMPC-5. In terms of cell quantity and morphology, OMPC-2.5 group possessed the best which is the closest to the control group.

**Figure 8.**
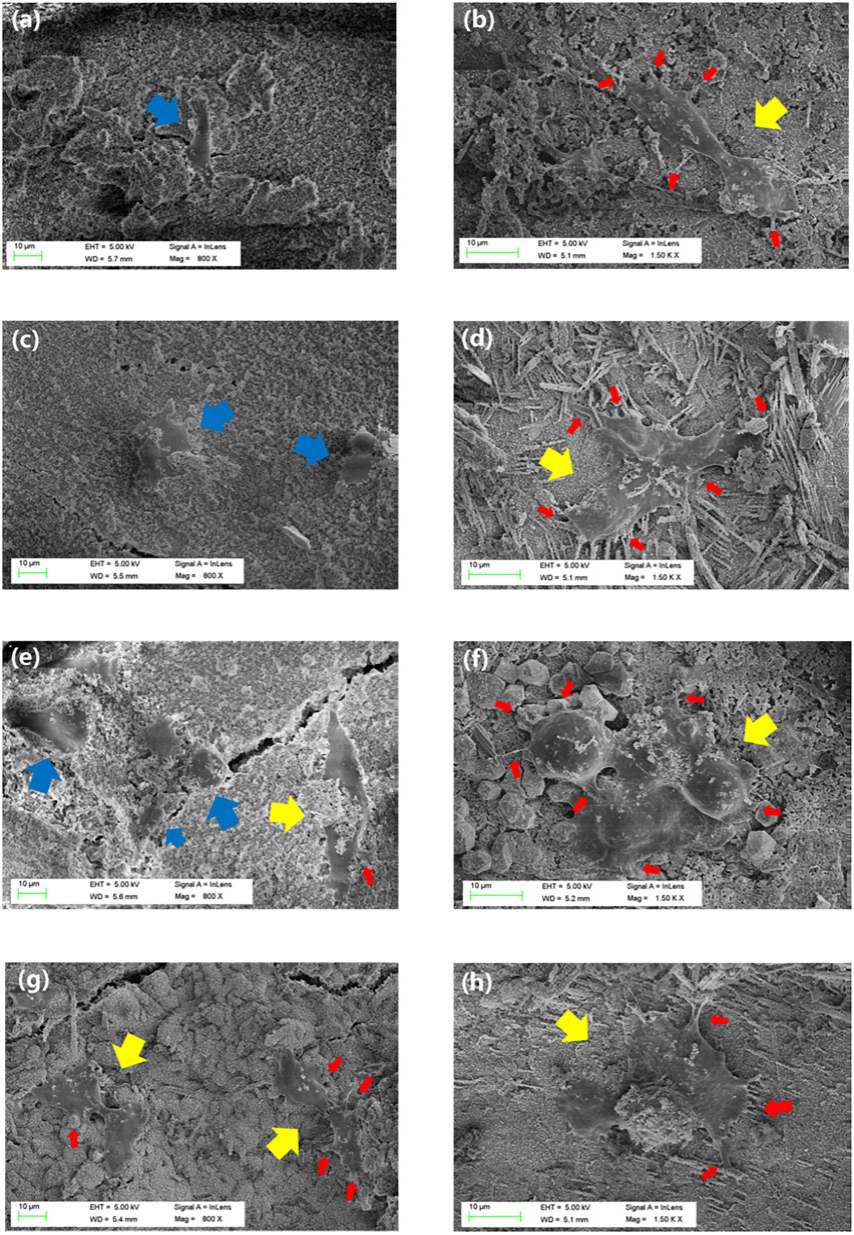
FESEM images of MC3T3-E1 cells seeded on OMPC-0 (**a**, **b**), OMPC-1 (**c**, **d**), OMPC-2.5 (**e**, **f**) and OMPC-5 (**g**, **h**) after 3 days (**a**, **c**, **e**, **g**) and 5 days (**b**, **d**, **f**, **h**). Blue and yellow arrows point the spherical and polygonal cells, respectively, red arrows represent the cell filopodia on the cement surface.

Field emission scanning electron micrographs of the MC3T3-E1 cells planted on the OMPC scaffolds are represented in Fig. 8. For OMPC-0 and OMPC-1, the cells were spherical after 3 days of culture. In response to subsequent incubation, they became elongated and adopted spindle-shaped configurations with large cell surfaces. However, cells on OMPC-2.5 and OMPC-5 already showed this changed appearance after Day 3 of culture, i.e. the cells showed quadrangular- or lozenge-shaped configurations. After Day 5, the cells on OMPC-2.5 showed strong adhesion, stretching, and aggregation and revealed some filopodia. The cells on OMPC-2.5 showed a much higher cell adhesion compared to other OMPC groups.

**Figure 9.**
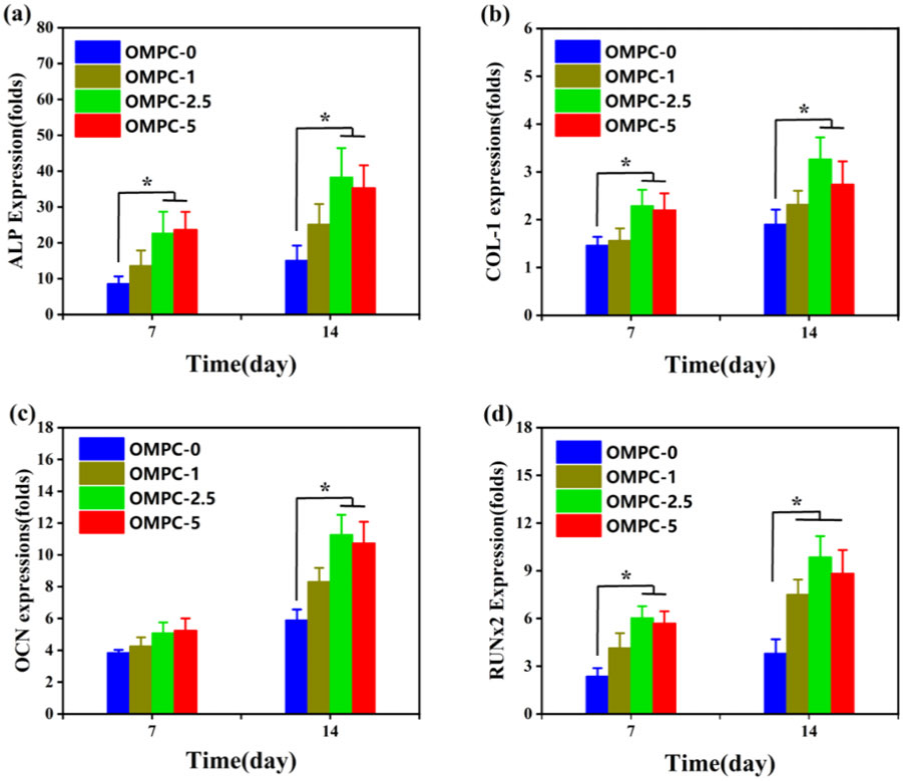
Osteogenesis-related differentiation of MC3T3-E1 cells cultured with OMPC scaffolds for 7 and 14 days: (**a**) ALP, (**b**) COL-1, (**c**) OCN and (**d**) Runx2 gene expression (*P < 0.05 vs. the OMPC-0 group).

The osteogenic differentiation results of MC3T3-E1 cells cultured with OMPC scaffolds are shown in Fig. 9. The expression of four osteogenic differentiation-related genes among all OMPC groups increased with increasing culture time and peaked at 14 days. Compared with MC3T3-E1 cells cultured on OMPC-0, cells cultured on OMPC-2.5 and OMPC-5 at Day 7 showed enhanced expression of ALP, COL-1 and Runx2. (*P* < 0.05). At Day 14, the OMPC-2.5 and OMPC-5 groups showed upregulated OCN expression, which consist with ALP, COL-1 and Runx2 (*P* < 0.05). Moreover, a significant difference was also found between the Runx2 expressions of OMPC-1 and OMPC-0 at Day 14.

## Discussion

K-struvite is a new-generation MPC that has acquired much attention in recent years. However, a lack of biological components limits its wide application [24]. In the present study, oxygen-carboxymethyl CS-doped K-struvite scaffolds (OMPCs) were successfully prepared. The hydration mechanism of this MPC system is reported to be a kind of fast acid−base reaction, and the reaction equations are listed below [25].

$$\mathrm{MgO}+\;{\mathrm{KH}}_{2}{\mathrm{PO}}_{4}+5H_{2}O\to 6\mathrm{MgKP}O_{4}\cdot 6H_{2}O$$

The XRD patterns of the OMPCs revealed unreacted MgO and the formation of MgKPO_**4**_·6H_**2**_O without any secondary hydration products involved; it is possible that O-CMC mainly acts as a microfiller in the OMPC paste. The possible reasons are as follows. First, the maximum added weight content of O-CMC was 5%, which is much lower than that of MgKPO_**4**_·6H_**2**_O in the final hydration products. Second, pure O-CMC showed two major diffraction peaks at approximately 2θ = 10.1° and 2θ = 20.4°, which is close to that of MgKPO4·6H2O (2θ  =  20.1°) (Supplementary Fig. S1). Third, the crystallinity of O-CMC was decreased after the carboxymethylation reaction, as Salama reported [26]. As indicated by the FTIR spectra of the OMPCs, no new bond vibration peaks were observed, and characteristic absorption peaks of carboxymethyl groups from O-CMC at 1620.2/cm (N−H), 1427.2/cm (−CH) and 1310.3/cm (−CH) were observed among all OMPC groups except for OMPC-0. The XRD data combined with the FTIR results indicated that O-CMC only worked as a retardant without participating in the formation of new hydration products.

To be more applicable for bone engineering applications, bone cement should have the properties of not only fast setting but also enough stiffness [27]. Regarding the setting time, the cement should set slowly enough to allow its handling by a clinician, but it should also set rapidly enough to ensure high initial strength; setting times ranging from 10 to 20 min are appropriate [27, 28]. In this study, a significant retarding effect was observed upon increasing the O-CMC content. The final setting time of OMPC-5 increased to 18 min, as shown in Fig. 1 (P < 0.05). The potential underlying mechanism is that O-CMC forms membranes coating the surfaces of MgO, thereby slowing its dissolution rate. Finally, O-CMC leads to a decrease in the rate of hydration product formation.

As a bone substitute, high initial strength is critical to prevent early-phase implant collapse [27]. As it affects the mechanical strength, the solid−liquid ratio was constant and set to 2 g/ml [29, 30]. As known in Fig. 1b, the compressive strength increased with increasing O-CMC content, and group OMPC-2.5 reached a maximum value of 33.8 MPa. This result is mainly attributed to O-CMC adsorbing deionized water to form a highly viscous film, which provides a barrier for crack propagation [31, 32]. This results in an increase in compressive strength. This phenomenon could be clearly observed in the FESEM images (Fig. 3), which show that OMPC-0 exhibited many cracks and pore structures, indicating its high brittleness, whereas in OMPC-5, the O-CMC prevented the growth of cracks and then formed a dense microstructure with less defects. However, the formation of crystal might be interfered by excess O-CMC and thereby damage the mechanical strength of OMPC-5 group. Thus, the compressive strength decreased with a further increase in the O-CMC content. Porosity is another vital parameter that must be considered because it significantly affects the mechanical properties of ceramics [33]. As exhibited in Fig. 1b, the porosity decreased with increasing O-CMC amount, and group OMPC-2.5 had the lowest porosity of 13.8%. The results suggested that the compressive strength of OMPC scaffolds was correlated with the porosity. The lower the porosity was, the higher the compressive strength of OMPC scaffolds had.

The pH of bone cement may influence the surface chemistry, thereby affecting the biological response, such as the proliferation and adhesion of cells [11]. In this study, the molar ratio of MgO to KH_2_PO_4_ was 1.3:1, but only a 1:1 ratio of MgO to KH_2_PO_4_ is required to form MgKPO_**4**_·6H_**2**_O, as described in the equation above. Excess MgO can not only act as an aggregate to enhance the compressive strength but also react with deionized water to produce OH^–^ [34]. We observed that the O-CMC incorporation neutralized the pH, possibly because of the existence of hydroxyl, amino and carboxyl groups. These functional groups improve the physical solubility and electric charge of O-CMC [35]. Therefore, O-CMC showed superior solubility at physiological pH and an increase in the surface positive charge after quaternization of CS, which resulted in the adsorption of OH^–^ in the OMPC system. The biodegradability of the bone cements is often considered a requirement [11]. This parameter is a good indication of the comparative dissolution rate between compositions, especially for MPCs, because the higher dissolution rates are one of the greatest advantages of MPCs [36]. The results from Fig. 5a revealed that the degradation rate was enhanced while adding O-CMC. This was possibly owing to the dissolution of saturated O-CMC. Regarding the Mg^2+^ release from the OMPC scaffolds, we detected that the Mg^2+^ release rate was correlated with the cement degradation rate. OMPC-5 had the highest ion concentrations of Mg^2+^ after 24 h (*P* < 0.05), and nearly 50% of Mg^**2+**^ was released during this period.

In general, biomaterials should interact actively with surrounding tissue and stimulate related cell proliferation, adhesion and differentiation [27, 37]. Cell culture assays were implemented here to verify the cytocompatibility of OMPC scaffolds. MC3T3-E1 cells proliferated with OMPC scaffolds, as exhibited by the CCK-8 assay, indicating ideally cellular responses to OMPC scaffolds. Moreover, the proliferation of MC3T3-E1 cells on OMPC-2.5 was much higher than that on the other groups, indicating that 2.5% concentration of O-CMC promoted cellular proliferation more effectively, which is consistent with the results of the cell morphology and the adhesion assay. Together with cell proliferation and adhesion, the ability of promoting differentiation on biomaterial surface is also an crucial aspect of cytocompatibility evaluation [27, 38]. Our results suggested that the expression levels of osteogenic differentiation-related genes with OMPC-2.5 group were significantly higher than those on OMPC-1 and OMPC-0 after 7 and 14 days. Yoshizawa *et al.* reported that different concentrations of free Mg^2+^ from MgSO_4_ significantly affected the cell function of human bone marrow stromal cells, and an Mg^2+^ concentration-dependent effect was observed [39, 40]. Therefore, considering all of the above experiments, we found that an appropriate addition of O-CMC enhanced the cytocompatibility of K-struvite, leading to an Mg^2+^-concentration dependent MC3T3-E1 behavior.

## Conclusion

In the present study, O-CMC-doped K-struvite composite scaffolds were successfully prepared by adding O-CMC at different mass fractions to the powder phase. The hydration reaction of K-struvite was found to be retarded in the presence of O-CMC. The phase composition and the surface morphology varied, which therefore affected the adhesion behavior of the MC3T3-E1 cells. *In vitro* experiments revealed that the Mg^2+^ release was related to the amount of O-CMC, which affected the degradation rate and the pH of the extracts of the OMPC samples. Additionally, *in vitro* cytocompatibility experiments showed that OMPC scaffolds improved the proliferation, adhesion and differentiation of MC3T3-E1 cells. In summary, MPC with a suitable content of O-CMC is promising for bone reconstruction.

## Supplementary data

Supplementary data are available at *REGBIO* online.

*Conflict of interest statement*. The authors have declared that no competing interest exists.

## Supplementary Material

rbaa048_Supplementary_Data
