# Supplementary material for: Enhancing the mechanical properties and cytocompatibility of magnesium potassium phosphate cement by incorporating oxygen-carboxymethyl chitosan
Source: Regen Biomater. 2020 Dec 3;8(1):rbaa048. doi: 10.1093/rb/rbaa048 (PMC7947597; doi:10.1093/rb/rbaa048)
Supplement: rbaa048_Supplementary_Data [file rbaa048_supplementary_data.docx]

**Supplementary**

Table S1. Primer sequences used in this study

| Gene | Primer sequence | |
| --- | --- | --- |
| ALP | Forward | 5′-ATCTTTGGTCTGGCCCCCATG-3′ |
|  | Reverse | 5′-AGTCCACCATGGAGACATTCTCTC-3′ |
| COL-1 | Forward | 5’-CGGACGAGGCAAGAGTTTCA-3’ |
|  | Reverse | 5’-TGTGAAATGCCACCTTTTGA-3’ |
| OCN | Forward | 5’-GAAGCCCAGCGGTGCA-3’ |
|  | Reverse | 5’-CACTACCTCGCTGCCCTCC-3’ |
| RUNX2 | Forward | 5’-ACGAGCTGAACAGGAACAACGT-3’ |
|  | Reverse | 5’-CACCAGCAAGAAGAAGCCTTTG-3’ |
| GAPDH | Forward | 5’-CCCCCAATGTATCCGTTGTG-3’ |
|  | Reverse | 5’-TAGCCCAGGATGCCCTTTAGT-3’ |





**Figure S1. XRD patterns of pure O-CMC**
